# Comparison of pressure resistance of double-rows and triple-rows circular stapler in rectal double stapling technique: In vitro study

**DOI:** 10.1097/MD.0000000000029600

**Published:** 2022-07-15

**Authors:** Junichi Mazaki, Kenji Katsumata, Ryutaro Udo, Tomoya Tago, Kenta Kasahara, Hiroshi Kuwabara, Masanobu Enomoto, Tetsuo Ishizaki, Yuichi Nagakawa, Akihiko Tsuchida

**Affiliations:** Department of Gastrointestinal and Pediatric Surgery, Tokyo Medical University, Tokyo, Japan.

## Abstract

**Background:**

Anastomotic leak after gastrointestinal anastomosis is a serious complication. Anastomotic failure depends on various parameters. The aim of our study was to evaluate the pressure resistance of a new device, EEA™ circular stapler with Tri-Staple™ technology 28 mm Medium/Thick (Triple-rows circular stapler; TCS) compared with EEA™ circular stapler with DST series™ technology 28 mm, 4.8 mm staples (double-rows circular stapler; DCS).

**Patients and methods:**

We performed 30 anastomoses (DSC: 15, TCS: 15) of DST with porcine colon model in vitro. We performed following 3 comparative experiences; Experiment 1: observation of staple shape with a colonoscopy, Experiment 2: comparison of the pressure resistance, Experiment 3: comparison of leakage points.

**Results:**

There was no hypoplasia of staples and the shapes were well-formed by colonoscopy. The leakage pressure of DCS was 19.6 ± 4.4 mm Hg (mean ± standard deviation) and that of TCS was 38.6 ± 10.2 mm Hg (mean ± standard deviation). There was a significantly difference between 2 groups (*P* < .001). 12 cases of DCS (80%) and 10 cases of TCS (66.7%) had leakages from Circular stapler point. 2 cases of DCS (13.3%) and 5 cases of TCS (33.3%) had leakages from Crossing points. Only 1 case of DCS had leakages from Dog ear point (6.7%). There was no significantly difference in leakage site between 2 groups (*P* = .195).

**Conclusions:**

TSC showed high pressure resistance during DST compared with that of DCS. It was suggested that TCS may contribute to the reduction of anastomotic leakage rate.

## 1. Introduction

Postoperative anastomotic leakage after gastrointestinal anastomosis can be a devastating complication,^[1,2]^ contributing to not only postoperative morbidity and mortality, but also local recurrence and poor functional outcomes.^[3]^ A successful anastomosis and subsequent healing depend on several factors, including the tension between the 2 connected portions of the gastrointestinal tract, a healthy blood supply to surrounding tissues, and the mechanical strength of the formed anastomosis.^[4–6]^ In particular, intraluminal pressure is one of the most influential risk factors for anastomotic leakage after rectal surgery.^[7–9]^ A diverting stoma is commonly used to decompress intraluminal pressure and avoid the risk of anastomotic leakage, but this method has several clinical disadvantages, including patient discomfort, inconvenience, and the need for later stoma closure surgery.^[10,11]^ Several preliminary studies have investigated the benefit of transanal decompression tubes for preventing anastomotic leakage.^[7–9]^ The beneficial roles of transanal tubes include endoluminal pressure reduction as well as fecal diversion, with a possible protective effect on anastomotic healing. While decompression of intraluminal pressure offers an additional approach, improving the pressure resistance of the anastomosis itself would be a fundamental solution.

The choice of surgical instruments greatly affects the safety of operations. There have been major advances in the development of medical devices for rectal surgery, in particular, circular staplers for rectal anastomosis with the double stapling technique (DST). Conventionally, the DST has been performed using a double-row circular stapler (DCS), but a new circular stapler introduced recently has a triple row of staples and is expected to improve pressure resistance. However, the effectiveness of this new triple-row circular stapler (TCS) has not been fully verified or compared relative to the conventional DST.

The present study aimed to compare the pressure resistance of TCS (EEA™ circular stapler with Tri-Staple™ technology, 28 mm Medium/Thick, Covidien, New Haven, CT) and DCS (EEA™ circular stapler with DST series™ technology, 28 mm, 4.8 mm, Covidien, New Haven, CT) using a porcine in vitro colon model.

## 2. Materials and Methods

### 2.1. Study design

**Figure 1.**
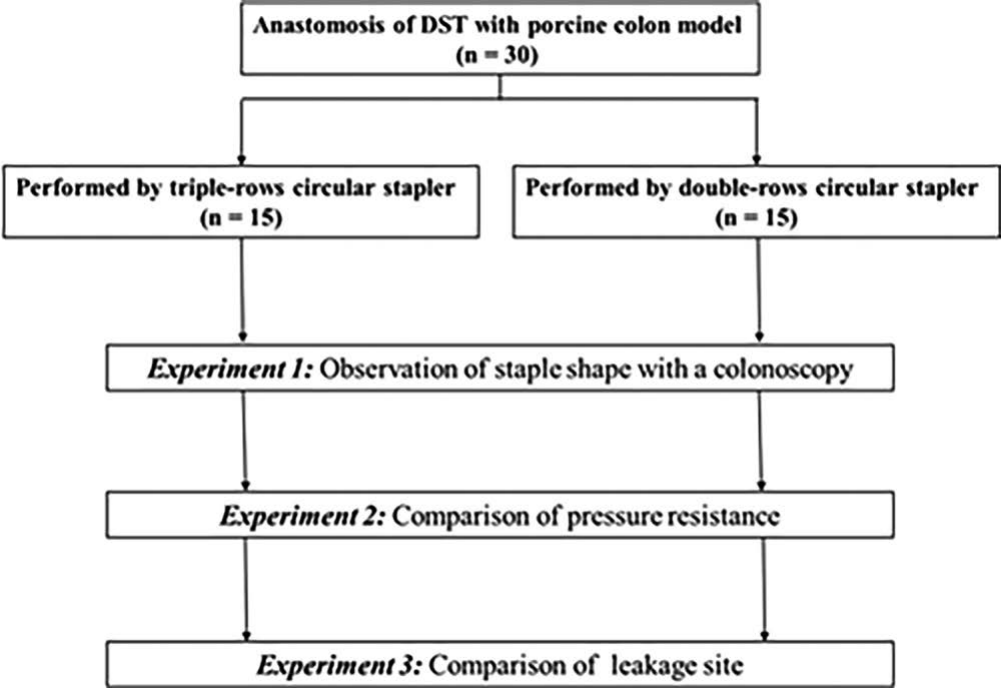
Study design and work flow.

We performed 30 anastomoses with the DST (DCS group, 15; TCS group, 15) using a porcine in vitro colon model. Colonoscopy was performed immediately after anastomosis, and anastomotic sites were observed and photographed from the inside (Experiment 1). Following this, comparisons of pressure resistance (Experiment 2) and leakage points (Experiment 3) between the DCS and TCS groups were performed. The study design and work flow are shown in Fig. 1. This study was approved by the Institutional Animal Ethics Committee of our institute.

### 2.2. Devices

**Figure 2.**
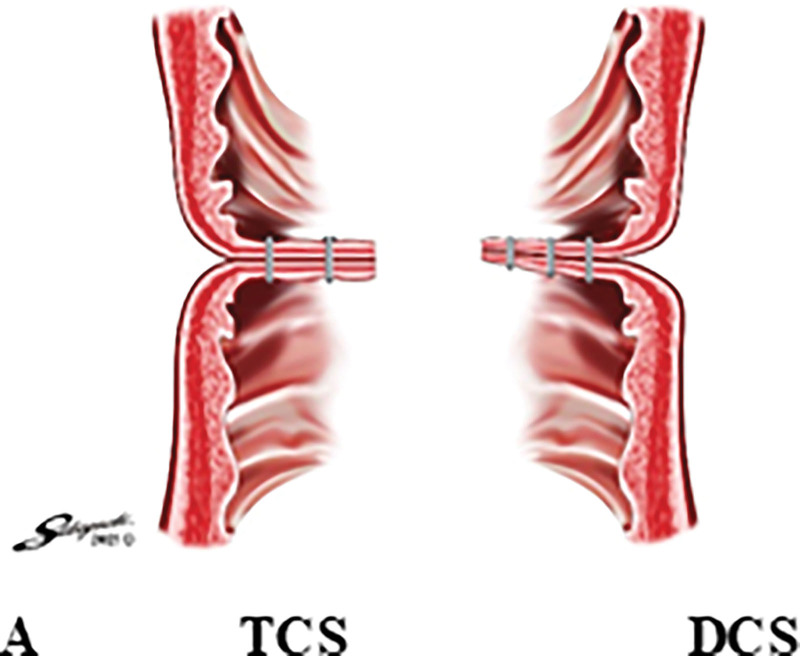
Characteristics of each circular stapler. A: Placement of each circular stapler, B: Height arrangement of each stapler.

The characteristics of each device are summarized in Table 1 and Figure 2. The EEA™ circular stapler with DST series™ technology 28 mm, 4.8 mm (Covidien, New Haven, CT) has a double row of staples of the same height, whereas the new device EEA™ circular stapler with Tri-Staple™ technology 28 mm Medium/Thick (Covidien, New Haven, CT) has a triple row of staples of different heights (gradually increasing from inside to outside) (Fig. 2). In this study, DCS of 28 mm and 4.8 mm and TCS of 28 mm and Medium/Thick were selected for caliber and staple height were selected, as they are most often used in clinical practice.

**Table 1:** Characteristics of TCS and DCS.

	Staple lines	Height (mm)	
		Before stapling	After stapling
TCS	3	3.0/3.5/4.0	1.2/1.5/1.75
DCS	2	3.5	1.5

DCS = double-row circular stapler, TCS = triple-row circular stapler.

### 2.3. Preparation of porcine colon samples

Porcine colons were obtained from a slaughterhouse (Tokyo Shibaura Zouki, Tokyo, Japan). Samples were immediately harvested from slaughtered animals and transported to the laboratory within 24 hours. Colons were cut into 20-cm sections for the anastomosis experiment.

### 2.4. DST anastomosis procedure

The center of each colon section was incised with a linear stapler (Signia™ Stapling System, Endo GIA™ 60-mm Articulating Medium/Thick Reload with Tri-Staple™ Technology, Covidien, New Haven, CT). The anvil of the circular stapler (i.e., DCS or TCS) was secured in place, and end-to-end anastomosis was performed with the DST. The rod of the circular stapler was inserted from the opposite side of the linear staple line, piercing the rectal stump near the linear staple line. When the anvil and rod were combined, we waited 20 seconds before firing. After firing, the stapler was held for 10 seconds and then released. All anastomoses were performed by an experienced surgeon.

### 2.5. Experiment 1: colonoscopic observation of staple shape

Colonoscopy was performed immediately after each anastomosis to observe the lumen using CF-Q260AI with EVIS LUCERA CV-260SL (Olympus, Tokyo, Japan).

### 2.6. Experiment 2: comparison of pressure resistance

**Figure 3.**
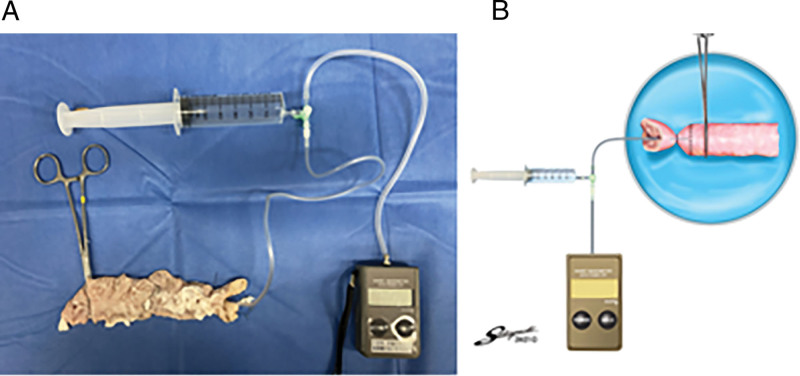
System used to examine leakage pressure and leakage points. A: Picture, B: Schema.

**Figure 4.**
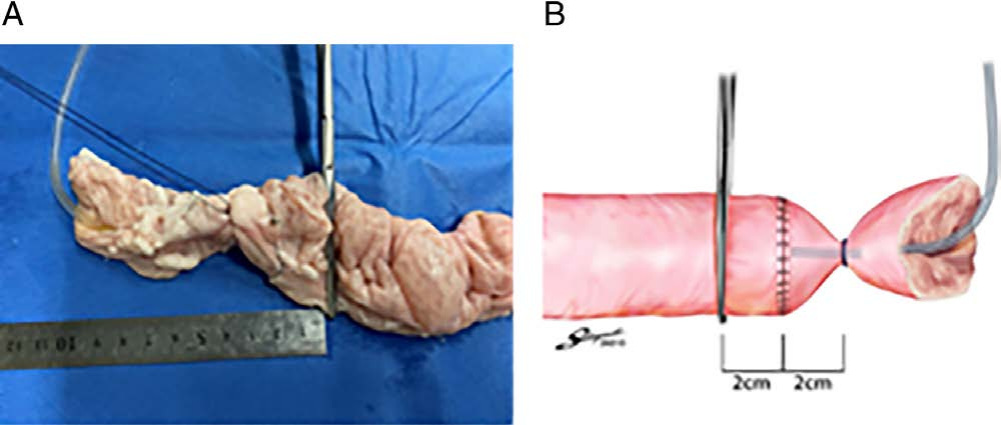
Unified clamping method to examine leakage pressure and leakage points. A: Picture, B: Schema.

A connecting tube was placed into the lumen of the transected colon. The tube was connected to an infusion pump and a pressure recorder (Handy manometer PG-100, Copal Electronics, Tokyo, Japan) via a pressure transducer (Fig. 3). In order to keep the experimental conditions constant for each anastomosis to measure pressure, the anal side of the anastomosis 2 cm was clamped with forceps, and the oral side of the anastomosis 2 cm and the tube were ligated with a thread (Fig. 4). In each case, the anastomotic site was immersed in water, and air was infused into the colon at a rate of 10 ml/min. Intraluminal pressure was continuously recorded. Leakage pressure was defined as the pressure at which air leakage from the anastomosis was initially observed.^[12]^

### 2.7. Experiment 3: comparison of leakage site

**Figure 5.**
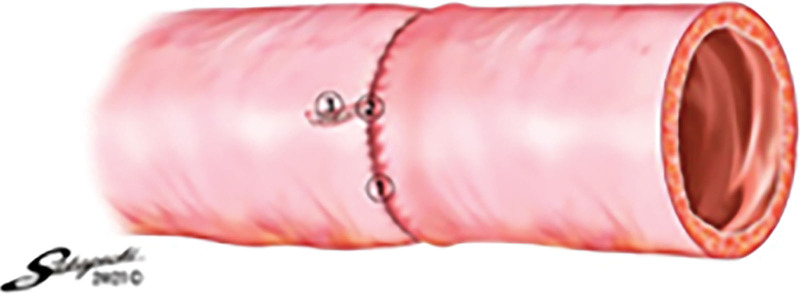
Schema of leakage sites. **①**: Circular stapler line (Circ), **②**: Crossing point of the circular stapler and linear stapler (Cros), **③**: Dog ear line (Dog).

Leakage sites were also recorded. In the porcine in vitro colon model, the leakage points were recorded as “circular stapler line (Circ),” “crossing point of the circular stapler and linear stapler (Cros),” and “Dog ear line (Dog)” (Fig. 5).

### 2.8. Statistical analysis

The primary outcome was pressure resistance. The Mann–Whitney U test was used to analyze discrete variables. Deviation scores were obtained to compare pressure resistance and leakage sites between the 2 devices. Statistical analyses were performed using SPSS version 25 software for Windows (IBM® SPSS® Statistics 25.0 Win® client version, IBM, Chicago, IL). *P* < .05 was considered statistically significant.

## 3. Results

### 3.1. Experiment 1: colonoscopic observation of staple shape

**Figure 6.**
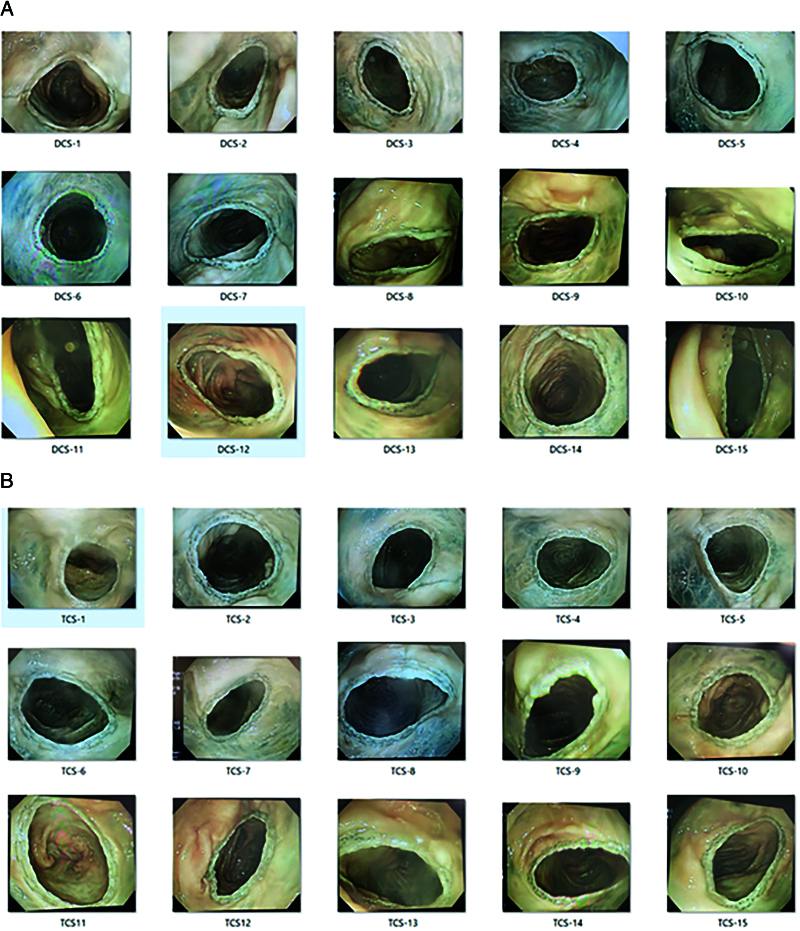
Colonoscopic images. A: Double-row circular stapler (DCS), B: Triple-row circular stapler (TCS).

Colonoscopic images are shown in Figure 6. In both DCS and TCS groups, no irregularity of staples formation was observed, and the shapes were well-formed.

### 3.2. Experiment 2: comparison of pressure resistance

**Figure 7.**
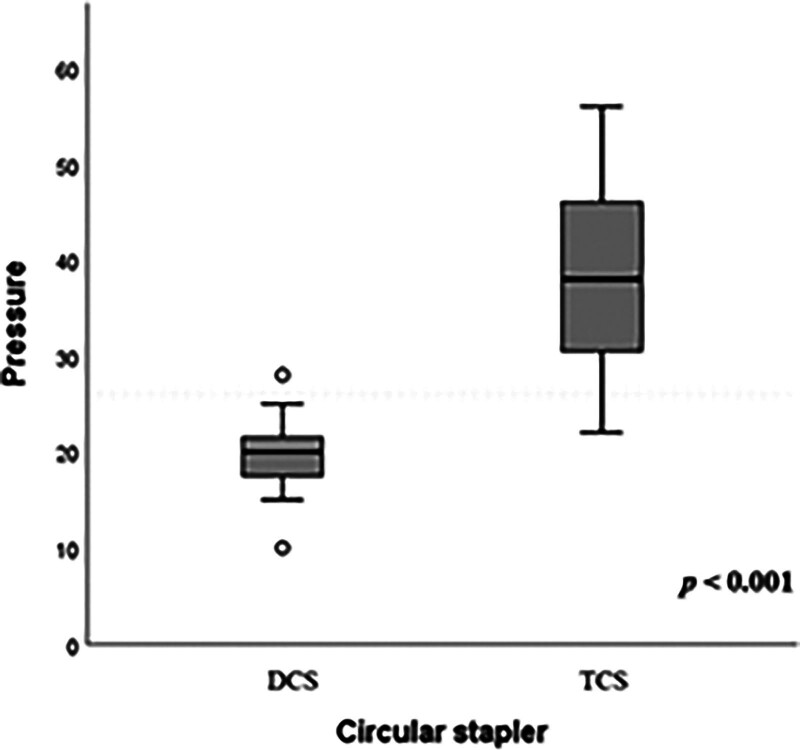
Comparison of leakage pressure.

The leakage pressure (mean ± standard deviation) was 19.6 ± 4.4 mm Hg for DCS and 38.6 ± 10.2 mm Hg for TCS (Table 2), showing a significant difference (*P* < .001) (Fig. 7). Deviation scores for each group are shown in Table 2. By showing deviation scores, it is possible to compare the pressure resistance between DCS and TCS.

**Table 2:** Leakage pressure and leakage site by stapler type.

DCS				TCS			
Case	Leakage pressure (mm Hg)	Deviation score	Site	Case	Leakage pressure (mm Hg)	Deviation score	Site
1	16	42	Circ	1	48	59	Cros
2	20	51	Circ	2	56	67	Circ
3	20	51	Circ	3	36	47	Circ
4	28	69	Circ	4	43	54	Circ
5	17	44	Circ + Cros	5	52	63	Circ
6	10	28	Circ	6	34	46	Circ + Cros
7	18	46	Circ	7	27	39	Circ
8	21	53	Circ	8	42	53	Circ + Cros
9	19	49	Circ + Circ	9	44	55	Circ + Circ
10	20	51	Circ	10	38	49	Circ + Cros
11	22	55	Dog	11	34	46	Circ
12	25	62	Circ	12	27	39	Circ
13	18	46	Circ + Cros	13	22	34	Circ
14	15	40	Circ	14	27	39	Circ + Cros
15	25	62	Circ + Circ	15	49	60	Circ

Circ = circular stapler line, Cros = crossing point of the circular stapler and linear stapler, DCS = double-row circular stapler, Dog = dog ear line, TCS = triple-row circular stapler.

### 3.3. Experiment 3: comparison of leakage sites

**Figure 8.**
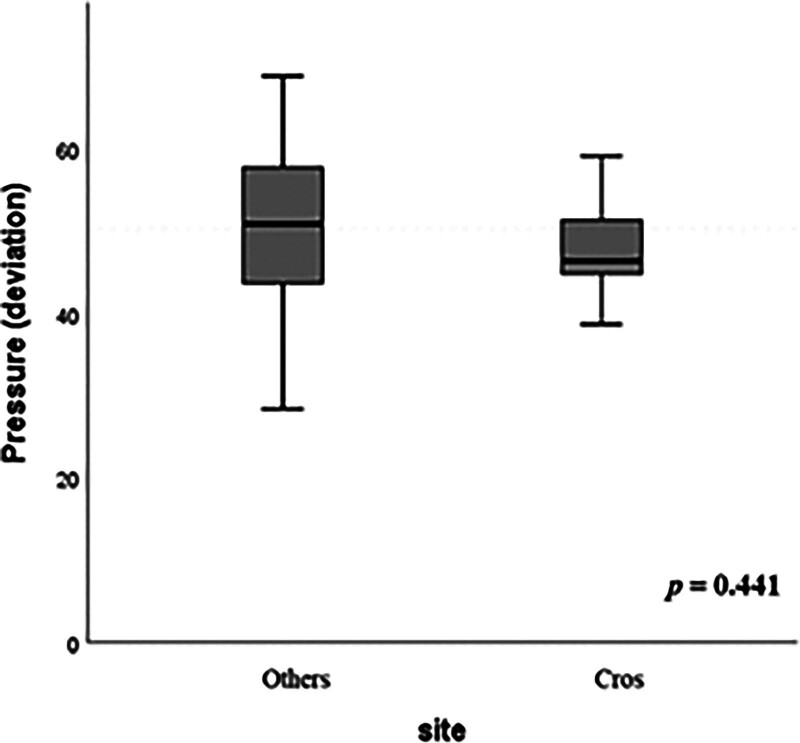
Comparison of leakage pressure and leakage sites.

The sites of anastomotic leakage are indicated in Table 2. In the DCS and TCS groups, 12 (80%) and 10 (66.7%) cases had leakage from the Circ point, respectively, and 2 (13.3%) and 5 (33.3%) cases had leakage from the Cros point, respectively. Only 1 (6.7%) case in the DCS group had leakage from the Dog point. Four (26.7%) and 5 (33.3%) cases in the DCS and TCS groups, respectively, had leakage from 2 points. Relationships between leakage site (Cros and others) and stapler type are shown in Table 3. No significant difference was observed in leakage site between DCS and TCS (*P* = .195). Based on deviation scores, there was no significant difference in leakage pressure by leakage site (*P* = .441) (Fig. 8).

**Table 3:** Relationships between leakage site (Cros and Others) and stapler type.

		Site		
		Cros	Others	Total
Stapler	DCS	2	13	15
	TCS	5	10	15
	Total	7	23	30

Cros = crossing point of the circular stapler and linear stapler, DCS = double-row circular stapler, TCS = triple-row circular stapler.

## 4. Discussion and Conclusions

Colorectal cancer is the second leading cause of cancer death in Japan. Surgical resection of colorectal cancer is the only curative treatment, for which the laparoscopic approach is now increasingly used. However, anastomotic leakage is a major problem in patients undergoing laparoscopic low anterior resection for rectal cancer, as this complication is associated with not only short-term but also long-term outcomes such as local recurrence and patient survival.^[13–18]^ Reducing anastomotic leakage has been recognized as a constant challenge for colorectal surgeons. The DST is the current, most prevalently used method for colorectal anastomosis.^[19]^ Since this method was reported by Knight and Griffen in 1980,^[20,21]^ its use in low anterior resections has been accepted by many surgeons. However, despite technical improvements and advances in equipment, the rate of leakage after anastomosis with the DST still remains at around 6% to 18% according to recent studies.^[16,19]^ In order to compare double- and triple-row circular staplers, it is necessary to make the anastomosis conditions for both constant. These conditions are the ones we use in clinical practice. Our reported anastomotic leakage rates under these conditions are in the single digits, which we consider acceptable.^[26]^

Anastomotic failure depends on various factors, including tissue thickness, collagen content, blood flow, improper selection of staple cartridges, ischemia, and tension.^[27,28]^ Anastomotic leakage appears to be closely related to the strength of a freshly created anastomosis. Many surgeons now perform blood flow examination with an indocyanine green (ICG) camera. The first clinical study reporting this method in colorectal surgery was conducted in 2010. Early randomized controlled trials in the field were reported in 2019 and 2020 by De Nardi et al^[29]^ and Jafari et al,^[30]^ respectively. In these prospective trials, there was no significant difference in the anastomotic leakage rate between the ICG and control groups. Moreover, there have been no reports on the use of blood flow examination in basic research. A greater leakage pressure is associated with a stronger anastomosis less than 1 week after surgery,^[31]^ suggesting that leakage pressure reflects the strength of the anastomosis. intraanal still pressure after rectal surgery is reported to reach 24 to 73 mm Hg.^[32,33]^ The leakage pressure is considered the most important factor in assessing the quality of a fresh intestinal suture line.^[34]^ Many surgeons interested in major leakage. While there is no evidence for a correlation between pressure resistance and grade of anastomotic leakage, we believe that anastomotic leakage itself and major leakage can be prevented by taking into account pressure resistance and taking additional measures such as the use of diverting stomas and transanal decompression tubes.

The importance of decompression after surgery has been demonstrated in a previous study. Xiao et al reported that, in a randomized prospective study, leakage was observed in 4% of patients with a transanal decompression tube, as compared to 9.6% in those without a decompression tube (*P* = .026).^[9]^ In that study, rectal resting pressure in patients with a transanal tube was reduced by roughly 7.5–10.5 mm Hg compared to patients without a transanal tube. In the present study, improved pressure resistance was observed in the TCS group (above 10 mm Hg on average), suggesting that a decrease in the rate of anastomotic leakage could be expected with TCS.

The DST has some drawbacks, including the lateral intersecting staple lines (the so-called dog ears), crossing point of the stapler, and multiple stapler firing, which could all lead to anastomotic leakage.^[35–38]^ Many surgeons may expect anastomotic leakage to commonly occur at the crossing point of the stapler. However, in previous reports, anastomotic leakage at the circular stapler point tended to be similarly or more commonly observed. In a study using a porcine in vitro model, Kawasaki et al observed leakage from the circular stapler line in 8 of 9 cases.^[39]^ Ikeda et al reported that anastomotic leakage from the circular stapler line was observed in about half of cases, with the remaining half showing leakage at the crossing point.^[40]^ Consistent with these reports, leakage from the circular stapler line was observed in more cases in the present study.

The absence of the crossing point does not appear to reduce the rate of anastomotic leakage. In previous studies, rates of anastomotic leakage did not significantly differ between the DST (with crossing point) and the single stapling technique (without crossing point).^[41–43]^ Moreover, given that the likelihood of the crossing point being the cause of anastomotic leakage is low, leakage from the circular stapler line is an important issue to address in order to reduce anastomotic leakage. On the other hand, simply tightening the circular stapler may further press and crush the already tightened colon wall due to purse-string sutures, and may thus result in the protrusion of a portion of the colon wall.^[40]^ Compared to DCS, which has a double row of staples of the same height, the more recent, improved TCS allows for gradual compression from the inside to the outside of the lumen, gradually releasing pressure outwards and thereby preventing severe compression damage.^[44–46]^ This mechanism may contribute to the greater pressure resistance of TCS.

This study has some limitations. First, we did not evaluate human specimens, as we were unable to acquire resected colons (which are very important for pathological diagnosis). Second, it is unclear whether the wall thickness of the porcine model matched the stapler height used. However, we selected the most commonly used height in clinical practice, and since endoscopic findings revealed no staple hypoplasia, our height selection was likely appropriate.

In conclusions, the pressure resistance of TCS is higher compared to DCS, suggesting that TCS may contribute to a reduction in the rate of anastomotic leakage.

## Author contributions

J.M. wrote the main manuscript text and T.T., K.K., H.K., M.E., T.I., acquired data for the work. All authors reviewed the manuscript. KK, YN, AT Drafted the work or revised it critically for important intellectual content.
